# Deep Neural Network Based Predictions of Protein Interactions Using Primary Sequences

**DOI:** 10.3390/molecules23081923

**Published:** 2018-08-01

**Authors:** Hang Li, Xiu-Jun Gong, Hua Yu, Chang Zhou

**Affiliations:** 1 School of Computer Science and Technology, Tianjin University, Nankai District, Tianjin 300072, China; lihang2499@126.com (H.L.); yuhua@tju.edu.cn (H.Y.); fujisyu@163.com (C.Z.); 2 Tianjin Key Laboratory of Cognitive Computing and Application, Nankai District, Tianjin 300072, China

**Keywords:** convolution neural networks, long short-term memory neural networks, protein–protein interaction, model generalization

## Abstract

Machine learning based predictions of protein–protein interactions (PPIs) could provide valuable insights into protein functions, disease occurrence, and therapy design on a large scale. The intensive feature engineering in most of these methods makes the prediction task more tedious and trivial. The emerging deep learning technology enabling automatic feature engineering is gaining great success in various fields. However, the over-fitting and generalization of its models are not yet well investigated in most scenarios. Here, we present a deep neural network framework (DNN-PPI) for predicting PPIs using features learned automatically only from protein primary sequences. Within the framework, the sequences of two interacting proteins are sequentially fed into the encoding, embedding, convolution neural network (CNN), and long short-term memory (LSTM) neural network layers. Then, a concatenated vector of the two outputs from the previous layer is wired as the input of the fully connected neural network. Finally, the Adam optimizer is applied to learn the network weights in a back-propagation fashion. The different types of features, including semantic associations between amino acids, position-related sequence segments (motif), and their long- and short-term dependencies, are captured in the embedding, CNN and LSTM layers, respectively. When the model was trained on Pan’s human PPI dataset, it achieved a prediction accuracy of 98.78% at the Matthew’s correlation coefficient (MCC) of 97.57%. The prediction accuracies for six external datasets ranged from 92.80% to 97.89%, making them superior to those achieved with previous methods. When performed on *Escherichia coli*, *Drosophila*, and *Caenorhabditis elegans* datasets, DNN-PPI obtained prediction accuracies of 95.949%, 98.389%, and 98.669%, respectively. The performances in cross-species testing among the four species above coincided in their evolutionary distances. However, when testing *Mus Musculus* using the models from those species, they all obtained prediction accuracies of over 92.43%, which is difficult to achieve and worthy of note for further study. These results suggest that DNN-PPI has remarkable generalization and is a promising tool for identifying protein interactions.

## 1. Introduction

Proteins often act through functions with their partners. These interacting proteins regulate a variety of cellular functions, including cell-cycle progression, signal transduction, and metabolic pathways [1]. Therefore, the identification of protein–protein interactions (PPIs) can provide great insight into protein functions, further biological processes, drug target detection, and even treatment design [2]. Compared to the experimental approaches, such as protein chips [3], tandem affinity purifications (TAP) [4], and other high-throughput biological techniques [5], computational methods for predicting PPIs are gaining greater exposure, as they are less labor-intensive and more efficient [6].

Currently, machine learning approaches dominate most of the computational methods for the prediction of PPIs [7]. Building a meaningful feature set and choosing corresponding machine learning algorithms are two key steps for successful predictions in traditional machine learning.

The feature set can be built by appropriate transformations of extracted features from the structures and sequences of proteins. Because of the low availability of structures, predicting PPIs using sequences alone has become one of the focus points [8,9,10]. Shen et al. [8] regarded any three continuous amino acids as a unit and calculated the frequencies of those conjoint triads as the features. Zhou [10] and Yang [11] used three local descriptors—composition, transition, and distribution—of amino acid sequences to predict PPIs. Guo et al. [9] constructed the feature vector of protein sequences with the autocovariance (AC) [9] method, which takes neighboring effects into account. In addition, there are several other feature representation methods, including the autocross-covariance (ACC) [9], multi-scale continuous and discontinuous (MCD) [12], and multi-scale local feature representation (MLD) [13] methods.

There are also some efficient classification algorithms for predicting PPIs, for instance, support vector machines [9,10,12] and their derivatives [14,15], random forests [13,16], neural networks [17], and gradient boosting decision trees [18]. It is noteworthy that each algorithm has its own preferences on corresponding feature sets. From this point of view, ensemble learning might yield more powerful predictors [6].

The recent advancements in deep learning technology have provided significant contributions in both scientific research and industry applications from speech and image recognition [19,20], computer vision [21], and decision making [22] to natural language processing [23]. These are composed of multiple linear and nonlinear transformations to model high-level abstractions by using a deep graph with multiple processing layers. The convolutional neural networks (CNNs) [24] and long short-term memory (LSTM) neural networks [25] are two typical architectures of deep learning.

Bioinformaticians are also devoting much effort to deep learning to solve their prediction problems ranging from peptide-MHC binding [26], cis-regulatory regions [27], and drug-induced liver injury [28] to DNA and RNA binding specificity [29,30]. Among these, most feed handcrafted features into the networks; few use features of the network itself from sequences alone. For instance, Sun et al. used a stacked autoencoder (SAE) [31] to study sequence-based PPI predictions using features from autocovariance and conjoint triad methods as the inputs of networks [32]. Pan et al. adopted a CNN and deep belief network (DBN) to discover RNA–protein binding motifs using different groups of features, that is, sequence, structure, clip-cobinding, region type, and motif features [33]. In our previous work, we developed a deep learning model for predicting DNA binding proteins only from sequences, in which the features were automatically learned by the networks themselves [34]. In this study, we further investigated the capability of auto feature engineering in the deep learning framework for predicting PPIs.

In this paper, we present a deep neural network framework (DNN-PPI) for predicting protein interactions. As for our previous work on predicting DNA binding proteins, one of highlights of DNN-PPI is that it only relies on raw data, without the need to manually extract features. Within the framework, the sequences of two interacting proteins are sequentially fed into the encoding, embedding, CNN, and LSTM layers. Then, a concatenated vector of the two outputs from the previous layer is wired as the input of the fully connected neural network. Finally, the Adam optimizer is applied to learn the network weights in a back-propagation fashion. The different types of features, including semantic associations between amino acids, position-related sequence segments (motif), and their long- and short-term dependencies, are captured in the embedding, CNN, and LSTM layers, respectively.

When the model was trained on Pan’s human PPI dataset, it achieved a prediction accuracy of 98.78% at the Matthew’s correlation coefficient (MCC) of 97.57%. The prediction accuracies for six external datasets ranged from 92.80% to 97.89%, making them superior to those achieved with previous methods. When performed on *Escherichia coli*, *Drosophila*, and *Caenorhabditis elegans* datasets, DNN-PPI obtained prediction accuracies of 95.949%, 98.389%, and 98.669%, respectively. The performances in cross-species testing among the four species above coincided in their evolutionary distances. However, when testing *Mus Musculus* using the models from those species, they all obtained over 92.43% prediction accuracies, which is difficult to achieve and worthy of note for further study.

## 2. Materials and Methods

### 2.1. Datasets

#### 2.1.1. Benchmark Dataset

We obtained raw data from Pan’s PPI dataset: http://www.csbio.sjtu.edu.cn/bioinf/LR_PPI/Data.htm [35]. The dataset contained 36,630 positive pairs and 36,480 negative pairs. The positive samples (PPIs) were from the human protein references database (HPRD) (2007 version), obtained by removing duplicated interactions (36,630 pairs remained). Negative samples (noninteraction pairs) were generated by pairing proteins found in different subcellular locations. After removing protein pairs with sequences of more than 1200 residues, the benchmark dataset contained 29,071 positive and 31,496 negative samples. We randomly selected 6000 (2943 positive and 3057 negative) samples as the hold-out testing set for model validation; the remainder were used as the training set. See Table 1 for details.

#### 2.1.2. Validation Datasets

In order to verify the generalization capability of the proposed method, we built several validation datasets from four well-known PPI data sources.

1. HPRD: The HPRD is a centralized repository for domain architecture, post-translational modifications, interaction networks, and disease associations in the human proteome. All the information in the HPRD was manually extracted from literature by expert biologists. The 2010 version of the HPRD dataset for protein interactions was downloaded.
2. DIP: The Database of Interacting Proteins (DIP) archives and evaluates experimentally determined interactions between proteins. All the interactions in the DIP are culled from peer-reviewed literature and are manually entered into the database by expert curators. The released version 20160430 was downloaded.
3. HIPPIE: The Human Integrated Protein–Protein Interaction Reference (HIPPIE) provides confidence-scored and functionally annotated human protein–protein interactions. The PPIs with confidence scores equal to or greater than 0.73 were regarded as “high quality” (HQ) data, while those with scores lower than 0.73 were regarded as “low quality” (LQ) data. Both HQ and LQ data of HIPPIE (version 2.0) were downloaded.
4. inWeb_inbiomap: This integrates eight large PPI databases and provides a scored human protein interaction network with severalfold more interactions and better functional biological relevance than comparable resources. We also distinguished between two types of PPI data: HQ data, whose confidence score was equal to 1, and LQ data for the rest. The newly released inWeb_inbiomap was downloaded.

We removed the protein pairs common to the benchmark dataset or having a sequence of more than 1200 amino acids from all of the downloaded datasets. Additionally, lower-redundancy versions were built by removing pairs with sequences sharing more than 40% sequence identity using the CD-HIT program. See Table 2 for details. It should be noted that datasets 1–6 contained only positive samples.

#### 2.1.3. Datasets from Other Species

We also used PPI datasets from other species, including *E. coli*, *Drosophila*, *C. elegans*, and *Mus Musculus*, to evaluate the performance of our model. The first three datasets were provided by Guo et al. (http://cic.scu.edu.cn/bioinformatics/predict_ppi/default.html) [9], all of which were built from the original DIP dataset. The protein pairs with an amino acid sequence of more than 1200 were removed from the datasets. The training and testing datasets were randomly extracted from the corresponding original datasets. The last dataset was downloaded from the Mint database. We removed the protein pairs common to the benchmark dataset or sharing a sequence of more than 1200 amino acids. It should be noted that this dataset had only positive samples. See Table 3 for details.

### 2.2. Methods

**Figure 1:**
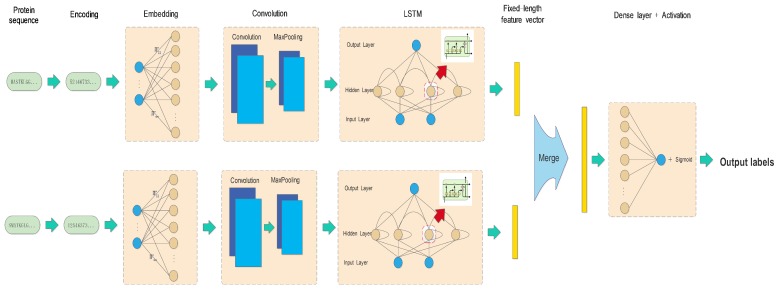
Architecture of the deep learning model.

**Architecture of the deep learning model**: Feature extractions and transformations are the most trivial tasks in the scenarios of traditional machine learning and most of deep learning. In this work, we made a simple encoding of amino acids in the protein sequence and then left the rest of the work to be done automatically by networks. Specifically, the two encoded sequences of interaction pairs were separately fed into layered networks, including embedding, CNN, and LSTM layers. Then, a concatenated vector of the two outputs from the previous layer was wired as the input of the fully connected neural network (dense layer). Finally, the Adam optimizer was applied to learn the network weights in a back-propagation fashion. The details of the proposed framework are shown in Figure 1.

The embedding layer acts as a nonlinear map, transforming the encoded digital vector into a numerical vector, which allows us to use continuous metric notions of similarity to evaluate the semantic associations between amino acids. The CNN layer consists of more convolutional layers, each followed by a max-pooling operation. The CNN can enforce a local connectivity of patterns between neurons of layers to exploit spatially local structures. Specifically, the CNN layer is used to capture nonlinear position-related features of protein sequences, for example, motifs, and enhances high-level associations with protein interactions. Enabling order dependence in sequence-based prediction problems to be captured, the LSTM network is used to learn short-term dependencies at the amino acid level and long-term dependencies at the motif level. After the layered network processing, the two outputs are merged into a feature vector as the input of a dense layer. The fully connected dense layer with dropout is used to detect the full associations between features with protein interaction functionality. The distance between the outputs of the dense layer and the true labels of paired proteins is measured by the binary cross-entropy. The network weights from the embedding layer to the dense layer are trained in a back-propagation fashion using the Adam optimizer.

The details are explained below for each processing layer.

**Protein sequence encoding**: In most sequence-based prediction problems, feature encoding is a tedious and critical task for constructing a statistical machine learning model. In order to demonstrate the powerful capability of feature learning in the deep learning model, we encode an amino acid by a natural number randomly. For a given protein sequence, a fixed-length digital vector is generated by replacing the amino acids with their corresponding encoders. If its length is less than “max_length”, we pad zeros to the front of the sequence.

**Embedding layer**: After the encoding, a protein sequence is converted to a sparse vector, as there are many zeros if its length is less than max_length. Furthermore, a protein residue often functions with its sequential and spatial neighbors. The simple encoding cannot reflect this type of relationship. Inspired by the “word2vec” model in natural language processing, we simulate protein sequences as documents, amino acids as words, and motifs as phrases. The embedding maps amino acids in a protein sequence to dense vectors. The semantic similarity of amino acids within the vector space is learned from large-scale sequences. This type of transformation allows us to use continuous metric notions of similarity to evaluate the semantic quality of individual amino acids. Embedding an amino acid can be done by multiplying the one-hot vector from the left with a weight matrix $W\in R^{d\times \left|V\right|}$, where |V| is the number of unique amino acids and *d* is the embedding size. Supposing that $v_{i}$ is the one-hot vector of an amino acid $x_{i}$ in a given protein sequence $x=x_{1}x_{2}\cdots x_{n}$, the embedding of $x_{i}$ can be represented as in Equation (1):

(1) $$e_{i}=Wv_{i}$$

The weight matrix is randomly initialized and updated in a back-propagation fashion. After the embedding layer, an input sequence can be presented by a dense matrix $E_{d\times n}=\left(e_{1},e_{2},\cdots ,e_{n}\right)$.

**Convolution layer**: CNNs are a type of feed-forward neural network and have been successfully applied to image recognition. Local connection detecting and weight sharing are two unique features of CNNs. The former allows us to discover local associations among features, while the latter can greatly reduce the computation complexity in training networks.

**Figure 2:**
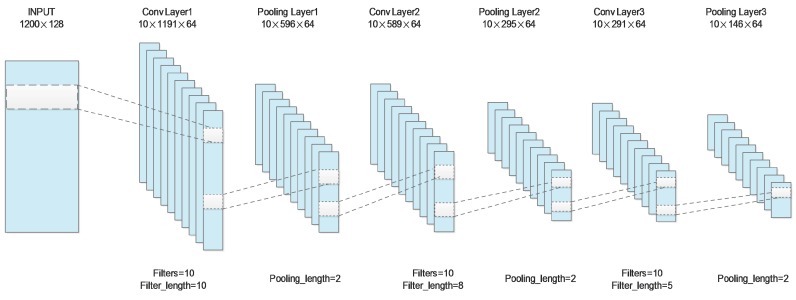
The architecture of convolutional neural network (CNN) layer.

DNN-PPI uses three layered CNNs, each of which that are followed by a max-pooling operation, to process embeddings of a protein sequence just as for an image; see Figure 2. For all the layers, we use the rectified linear unit (ReLU) as an activation function, and the length of the max-pooling is set to 2. The first convolution layer applies 10 filters with a filter length of 10 to the input matrix and outputs a 10×1191×64 feature map. After max-pooling, the hidden feature map with a size of 10×596×64 is used as input to the second layer. Repeating the above steps twice (the lengths of filters in the second and third layers are set to 8 and 5, respectively), we finally obtain a 10×146×64 feature map.

**Figure 3:**
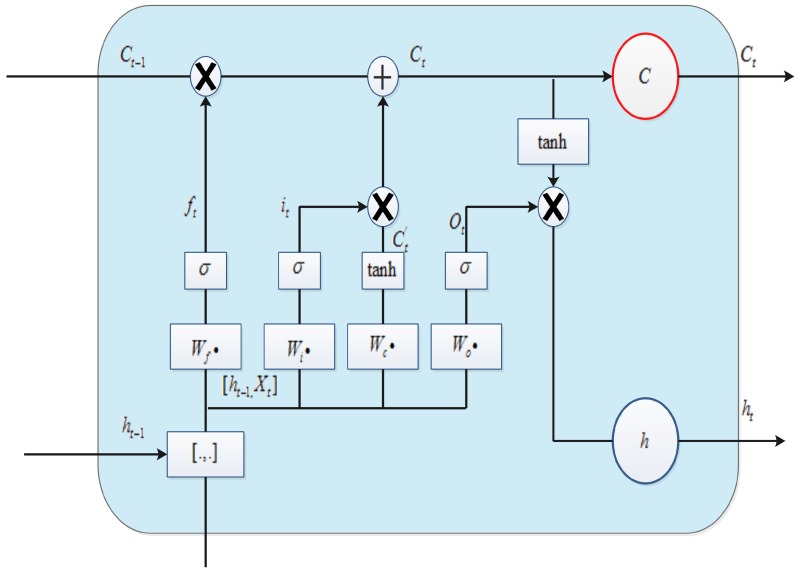
Long short-term memory cell.

**LSTM layer**: Because of the problem of vanishing and exploding gradients in the recurrent neural network (RNN) model, it is difficult to learn long-term dynamics. As a variant of RNN, LSTM provides a solution by incorporating memory units that allow the network to learn when to forget previous hidden states and when to update hidden states according to the given information. It uses purpose-built memory cells to store information; see Figure 3 for a typical LSTM cell [36].

The components of a LSTM cell are explained by Equations (2)–(6), where σ represents the logical sigmoid function and *i*, *f*, *o*, and *c* represent the input gate, forget gate, output gate, and cell and cell-input activation vectors, respectively. All of these are the same size as the hidden vector *h*, for which $W_{hi}$ is the hidden-input gate matrix, $W_{xo}$ is the input–output gate matrix, and so on. The weight matrices from the cell to gate vectors (e.g., $W_{ci}$) are diagonal; thus an element *m* in each gate vector only receives input from element *m* of the cell vector. The bias terms (which are added to *i*, *f*, *c*, and *o*) are omitted for clarity.

(2) $$i_{t}=\sigma \left(W_{xi}x_{t}+W_{hi}h_{t-1}+W_{ci}c_{t-1}+b_{i}\right)$$

(3) $$f_{t}=\sigma \left(W_{xf}x_{t}+W_{hf}h_{t-1}+W_{cf}c_{t-1}+b_{f}\right)$$

(4) $$c_{t}=f_{t}c_{t-1}+i_{t}\tanh\left(W_{xc}x_{t}+W_{hc}h_{t-1}+b_{c}\right)$$

(5) $$o_{t}=\sigma \left(W_{xo}x_{t}+W_{ho}h_{t-1}+W_{co}c_{t}+b_{o}\right)$$

(6) $$h_{t}=o_{t}\tanh\left(c_{t}\right)$$

**Dense layer**: The outputs of the LSTM layer for the two sequences of interaction pairs are concatenated into a vector as the input of a fully connected neural network. In general, a sigmoid function demonstrates mathematical behaviors, such as being real-valued, being differentiable, having a non-negative or -positive first derivative, having one local minimum, and having one local maximum. Thus, in this work, we used such a function as the activation function of the network: Equation (7).

(7) $$o=sigmoid\left(S_{3}\right)=1/\left(1+e^{-S_{3}}\right)$$

A loss function measures how well a machine learning model fits empirical data. In this work, binary cross-entropy was used as the loss function; see Equation (8):

(8) binary_crossentropy(t,o)=−(t(log(o)+(1−t)(log(1−o))),

where *t* and *o* represent the target and the output, respectively.

Finally, the weights of networks are updated iteratively using the Adam optimizer, which combines the advantages of adaptive gradient and root-mean-square propagation algorithms.

The whole procedure is implemented in the Keras framework, a minimalist and highly modular neural network library. Keras is written in Python and is capable of running on top of either TensorFlow or Theano. It was developed with a focus on enabling fast experimentation and is supported on both CPUs and GPUs.

## 3. Results

In this section, we first show the performances of DNN-PPI on the training set of the benchmark dataset via 5-fold cross-validations. The best model among the 5-fold runs was used to test the hold-out set, and its performance comparisons with the state-of-the-art PPI predictors are also shown. A full model trained by the whole benchmark dataset was then applied to the six validation datasets and their low-redundancy versions. Finally, various cross-species testing experiments were designed for further evaluation of the generalization of DNN-PPI.

All the experiments used the same parameters. The input parameters and output sizes of each layer are shown in Table 4.

### 3.1. Evaluation Criteria

In order to evaluate the performances of the proposed method, we used the following evaluation metrics: accuracy (ACC), recall, precision, F-score, and Matthew’s correlation coefficient(MCC), which are defined by Equations (9)–(13):

(9) $$Accuracy=\frac{TP+TN}{TP+TN+FP+FN},$$

(10) $$Recall=\frac{TP}{TP+FN}$$

(11) $$Precision=\frac{TP}{TP+FP},$$

(12) $$F-Score=2\times \frac{Recall\times Precision}{Recall+Precision}$$

(13) $$MCC=\frac{TP\times TN-FP\times FN}{\sqrt{\left(TP+FN)\times (TN+FP)\times (TP+FP)\times (TN+FN\right)}},$$

where *TP*, *TN*, *FP*, and *FN* represent the numbers of true-positive, true-negative, false-positive, and false-negative samples, respectively.

### 3.2. Training and Validation on the Benchmark Dataset

We trained five models via 5-fold cross-validation on the benchmark training set. Then, the best model was used to predict the hold-out testing set.

The results are shown in Table 5 (to make the values comparable, we removed the % sign and used real values for fractal measures). All the values of the five measures were higher than 0.97 across the five runs. The averages of the five measures were 0.9896, 0.9901, 0.9882, 0.9891, and 0.9791. The fifth run achieved the best model performance, with an accuracy of 0.9941, recall of 0.9963, precision of 0.9915, F-score of 0.9939, and MCC of 0.9883. For testing the hold-out set, the best model achieved an accuracy of 0.9878, recall of 0.9891, precision of 0.9861, F-score of 0.9876, and MCC of 0.9757. These results show that DNN-PPI recognized PPIs with remarkable reliability and generalization for the benchmark dataset.

We also performed four groups of experiments with different proportions of the training and testing sets over the whole benchmark dataset, and the results are shown in Table 6. It can be seen that all four performance metrics were higher than 0.9672 and that there were no notable performance differences using different sizes of the training sets.

We also compared the performance of the best model on the hold-out testing set with those of the combinations of state-of-the-art feature extraction methods and classification algorithms. The feature extraction methods included 188D [37], QuaLitative Characteristic (QLC) and QuaNtitative Characteristic (QNC) features based methods [6]. SVM, random forests, GBDT, and SAE were used as classification algorithms. It should be noted that SAE as a deep learning model has been used for predicting PPIs in the literature [32].

As shown in Table 7, DNN-PPI outperformed nearly 1% of the best methods (GBDT and QNC). Compared with SAE, DNN-PPI performed significantly better, with 3.4% higher accuracy. Therefore, DNN-PPI has competitive performance against the existing methods for predicting PPIs.

### 3.3. Generalization Performances on the Validation Datasets

Generalization and overfitting are two important and closely related topics in the field of machine learning. Generalization is a term used to describe a model’s ability to react to new data, while overfitting refers to a model that fits training data too well. Although various techniques from both the dataset partition and algorithm design perspectives have been proposed, the problem remains because of future unseen data never being predictable. Case-by-case verification is still the most practical way for testing a model’s generalization.

Here, six external well-curated human PPI datasets were used to validate the generalization of DNN-PPI. Four models, DNN-PPI, SAE, GBDT, and the method of Pan et al., were trained on the whole benchmark dataset. SAE was downloaded from GitHub, provided by [32]; GBDT with QNC and QLC features was from our previous work; and the method of Pan et al. is performed by their online server (http://www.csbio.sjtu.edu.cn/bioinf/LR_PPI). Because the sizes of the last three datasets exceeded the limitations of Pan’s server, the corresponding results are filled with blanks; see Table 8.

DNN-PPI achieved 94.26% average accuracy over all six datasets, which was higher by nearly 10% and 1% than the best results of the other deep learning and traditional machine learning methods. The better performance of GBDT is partly explained by its intensive feature engineering. The fact that all the models worked best on the 2010 HPRD dataset shows the HQ of PPIs in this dataset. Additionally, all the models worked better in the HQ datasets than in their LQ versions: HIPPIE and inWeb_inbiomap. This further proves that data quality is an important factor for the success of machine learning.

The redundant sequences in these datasets may have resulted in pseudo performances in testing. We tested DNN-PPI on their low-redundancy versions. As shown in Table 9, the accuracies decreased by 0.0324, 0.0131, and 0.0188 on the first three datasets, while they increased by 0.0074, 0.0104, and 0.0051 on the last three datasets. There was no significant difference between the two versions of the datasets.

### 3.4. Performances on Other Species and Cross-Species Validations

We also tested the performances of DNN-PPI on three other species, *E. coli*, *Drosophila*, and *C. elegans*, each of which were provided by Guo et al. [9]. For each species, the positive and negative samples were mixed, and then 1/10 of them were randomly selected as a testing set; the remainder were used as the training set. The same settings were applied to SAE and Guo’s methods.

As shown in Table 10, the average values of recall, precision, MCC, F-score, and accuracy of DNN-PPI were 0.9637, 0.9888, 0.9536, 0.9761, and 0.9766. The accuracies on all three datasets were higher than those for SAE and Guo’s methods, with more than 3% and 1% increases on average. These results demonstrate the power of DNN-PPI for predicting PPIs in different species.

To validate the performances of cross-species testing, we trained DNN-PPI on the full datasets of the benchmark, *E. coli*, *Drosophila*, and *C. elegans* and then used the models to predict results for other species, including *Mus Musculus*.

As shown in Table 11, the maximum and minimum accuracies for cross-testing among humans and *E. coli*, *Drosophila*, and *C. elegans* species were 0.5267 and 0.4585. These lower prediction accuracies suggest that the four species have long evolutionary relationships and that their PPIs are far from those of others. The model of humans achieved an accuracy of 98.35% for testing *Mus Musculus*, as the species have a close genetic relationship. However, it is a rare situation that models of other species work also very well on *Mus Musculus*, as they have long distances in terms of genetic evolution.

## 4. Discussion

The number of CNN layers is of crucial importance for the discriminant power in the deep learning field. For instance, Hou et al. designed the DeepSF consisting of 10 1D convolution layers for mapping protein sequences into folds [38]. On the challenging ImageNet task, the layers of CNNs were exploited, numbering from 16 [39] to 30 [40].

**Figure 4:**
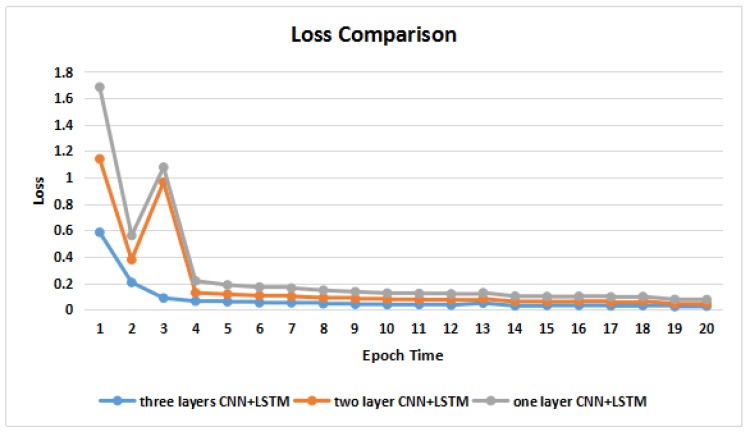
Loss comparisons across different models.

**Figure 5:**
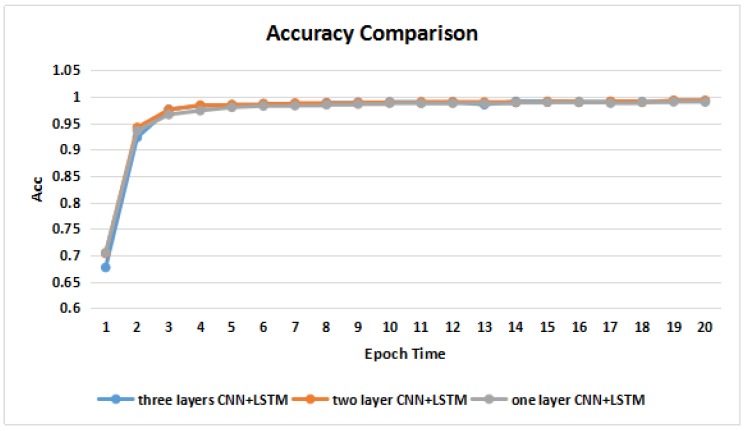
Accuracy comparisons across different models.

To verify the convergences and accuracies of the models with different layers of CNNs, we conducted two additional experiments with one and two layers of CNNs. As shown in Figure 4 and Figure 5, the model with a higher number of CNN layers had a faster convergence speed in terms of the loss value. There was no significant difference in their accuracies among the three models trained over epoch times.

There are also several other architectures of DNNs, including DBNs, RNNs, generative adversarial networks (GANs), and SAEs, a few of which have been applied to predict PPIs. The latest work done by Sun et al. [32] used a SAE for identifying protein interactions from sequences. Compared to the simple one-hot encoding in DNN-PPI, the protein sequences in SAE are coded by autocovariance and conjoint triad extraction methods. The performance comparisons are discussed in the results section.

The most recent work for predicting PPIs using sequences was presented by Wang et al. [41]. They encoded protein sequences by combing the continuous and discrete wavelet transforms and used a weighted sparse-representation-based classifier for predicting. Its average accuracy and MCC on the DIP (5594 positive and 5594 negative samples) of human PPIs were 98.92% and 98.93%, which were similar to those of DNN-PPI on the benchmark dataset. When testing 312 pairs of *Mus Musculus* using the model trained on yeast samples, they achieved 94.23% accuracy, while the DNN-PPI models of four species achieved the best (98.35%) accuracy and average accuracy of 95.67% for all 22,870 pairs.

## 5. Conclusions

In this paper, we present a DNN framework (DNN-PPI) for predicting protein interactions only using primary sequences. It consists of five layered building blocks, including encoding, embedding, CNN, LSTM, and dense layers. After simple one-hot encoding sequences of interaction pairs separately, semantic associations between amino acids are learned in the embedding block. The three layers of CNNs and LSTM networks allow for mining the relationships between amino acid fragments in terms of local connectivity and long-term dependence. Finally the learned features are fed into a fully connected dense layer to make predictions. All the parameters of the networks are trained in a back-propagation fashion using the Adam optimizer.

DNN-PPI outperforms traditional machine learning and other deep learning methods with remarkable performance and reliability on the benchmark dataset. The independent testing on six validation datasets and cross-species testing suggested that DNN-PPI has competitive generalization capability for predicting PPIs. The proposed deep learning framework would have many other potential applications, such as predicting protein–DNA/RNA interactions, drug target interactions, their interaction residues, and so on.

## Figures and Tables

**Table 1:** Benchmark dataset.

Dataset	Positive Samples	Negative Samples	Total
Benchmark set	29,071	31,496	60,567
Training set	26,128	28,439	54,567
Hold-out test set	2943	3057	6000

**Table 2:** Validation datasets.

Dataset	2010 HPRD	DIP	HIPPIE HQ	HIPPIE LQ	inWeb_inbiomap HQ	inWeb_inbiomap HQ
Positive samples	8008	4514	25,701	173,343	128,591	368,882
Low-redundancy	2413	1276	3035	5587	2546	5358

**Table 3:** Protein–protein interaction (PPI) datasets for four species.

Species	Dataset	Positive Samples	Negative Samples	Total
*E. coli*	Original set	6680	6881	13,561
	Training set	6012	6193	12,205
	Testing set	668	688	1356
*Drosophila*	Original set	19,133	18,449	37,582
	Training set	17,220	16,593	33,813
	Testing set	1913	1856	3769
*C. elegans*	Original set	3696	3763	7459
	Training set	17,220	16,593	33,813
	Testing set	1913	1856	3769
*Mus Musculus*	Original set	22,870	—	22,870

**Table 4:** The parameters and output sizes of each layer.

Layer	Parameters	Output_Size of Protein A	Output_Size of Protein B
Input	Sentence_length = 1200	(128,1200)	(128,1200)
	Batch_size = 128		
Embedding layer	Input_dim = 23	(128,1200,128)	(128,1200,128)
	Output_dim = 128		
Convolution layer 1	Filters = 10	(128,1191,64)	(128,1191,64)
	Filter_length = 10		
	Activation = relu		
MaxPooling	Pooling_length = 2	(128,596,64)	(128,596,64)
Convolution layer 2	Filters = 10	(128,589,64)	(128,589,64)
	Filter_length = 8		
	Activation = relu		
MaxPooling	Pooling_length = 2	(128,295,64)	(128,295,64)
Convolution layer 3	Filters = 10	(128,291,64)	(128,291,64)
	Filter_length = 5		
	Activation = relu		
MaxPooling	Pooling_length = 2	(128,146,64)	(128,146,64)
LSTM layer	Output_size = 80	(128,80)	(128,80)
Merge layer	Mode = concat	(128,160)	
Output	Activation = sigmoid	(128,1)	

**Table 5:** Performances of deep neural network/protein–protein interaction (DNN-PPI) framework on the benchmark dataset.

Test Set	Accuracy	Recall	Precision	F-Score	MCC
1	0.9853	0.9845	0.9849	0.9847	0.9706
2	0.9877	0.9876	0.9865	0.9871	0.9754
3	0.9916	0.9909	0.9913	0.9911	0.9831
4	0.9892	0.9911	0.9867	0.9889	0.9784
5	0.9941	0.9963	0.9915	0.9939	0.9883
Hold-out	0.9878	0.9891	0.9861	0.9876	0.9757

**Table 6:** Performances with different proportions of training and testing sets on the benchmark dataset.

Training/Testing	Accuracy	Recall	Precision	F-Score	MCC
0.70/0.30	0.9846	0.9796	0.9883	0.9839	0.9693
0.75/0.25	0.9870	0.9864	0.9867	0.9865	0.9741
0.80/0.20	0.9836	0.9768	0.9889	0.9828	0.9672
0.85/0.15	0.9849	0.9821	0.9864	0.9843	0.9698

**Table 7:** Performance comparisons on the hold-out test set.

Method		Accuracy	Average
188D	SVM	0.9468	0.9645
	RF	0.9701	
	GBDT	0.9767	
QLC	SVM	0.9497	0.9658
	RF	0.9701	
	GBDT	0.9775	
QNC	SVM	0.9582	0.9686
	RF	0.9695	
	GBDT	0.9782	
QNC + QLC	SVM	0.9758	0.9751
	RF	0.9716	
	GBDT	0.9778	
SAE		0.9538	0.9538
DNN-PPI		0.9878	0.9878

**Table 8:** Accuracy comparisons on validation datasets.

Dataset Name	Samples	DNN-PPI	SAE	GBDT	Pan et al.
2010 HPRD	8008	0.9789	0.9205	0.9663	0.8816
DIP	4514	0.9433	0.8773	0.9465	0.8872
HIPPIE HQ	25,701	0.9608	0.8623	0.9415	0.8301
HIPPIE LQ	173,343	0.9340	0.8105	0.9180	—
inWeb_inbiomap HQ	128,591	0.9307	0.8512	0.9284	—
inWeb_inbiomap LQ	368,882	0.9280	0.8187	0.9028	—

**Table 9:** DNN-PPI’s accuracy on low-redundancy versions of validation datasets.

Dataset	Samples	ACC
2010 HPRD LR	2413	0.9465
DIP LR	1276	0.9302
HIPPIE HQ LR	3035	0.9420
HIPPIE LQ LR	5587	0.9414
inWeb_inbiomap HQ LR	2546	0.9411
inWeb_inbiomap LQ LR	5358	0.9331

**Table 10:** Performance comparisons on datasets for other species.

Species	Recall	Precision	MCC	F-Score	Accuracy	SAE ACC	Guo et al. ACC
*E. coli*	0.9416	0.9752	0.9194	0.9581	0.9594	0.9323	0.9528
*Drosophila*	0.9686	0.9995	0.9681	0.9837	0.9838	0.9348	0.9623
*C. elegans*	0.9810	0.9918	0.9732	0.9864	0.9866	0.9786	0.9732

**Table 11:** Performances on the cross-species validations.

Training Set	Test Set	Accuracy
Benchmark dataset	*Mus Musculus*	0.9835
	*C. elegans*	0.5267
	*Drosophila*	0.5205
	*E. coli*	0.4754
*C. elegans*	*Mus Musculus*	0.9243
	Benchmark dataset	0.4886
	*Drosophila*	0.5230
	*E. coli*	0.4812
*Drosophila*	*Mus Musculus*	0.9713
	Benchmark dataset	0.4803
	*C. elegans*	0.5147
	*E. coli*	0.4924
*E. coli*	*Mus Musculus*	0.9475
	Benchmark dataset	0.4563
	*C. elegans*	0.4585
	*Drosophila*	0.4871
